# Hepcidin and ferritin levels as markers of immune cell activation during septic shock, severe COVID-19 and sterile inflammation

**DOI:** 10.3389/fimmu.2023.1110540

**Published:** 2023-01-27

**Authors:** Marcela Hortová-Kohoutková, Monika Skotáková, Isaac G. Onyango, Miriam Slezáková, Roman Panovský, Lukáš Opatřil, Peter Slanina, Marco De Zuani, Ondřej Mrkva, Ivana Andrejčinová, Petra Lázničková, Martina Dvončová, Alexandra Mýtniková, Vaughn Ostland, Michal Šitina, Gorazd B. Stokin, Vladimír Šrámek, Marcela Vlková, Martin Helán, Jan Frič

**Affiliations:** ^1^ International Clinical Research Center, St. Anne’s University Hospital, Brno, Czechia; ^2^ 1st Department of Internal Medicine/Cardioangiology, Faculty of Medicine, Masaryk University, Brno, Czechia; ^3^ Institute of Clinical Immunology and Allergology, Faculty of Medicine, Masaryk University, Brno, Czechia; ^4^ Department of Biology, Faculty of Medicine, Masaryk University, Brno, Czechia; ^5^ Department of Anesthesiology and Intensive Care, Faculty of Medicine, Masaryk University, Brno, Czechia; ^6^ Intrinsic Lifesciences, La Jolla, CA, United States; ^7^ Celica BIOMEDICAL, Ljubljana, Slovenia; ^8^ Division of Neurology, University Medical Centre, Ljubljana, Slovenia; ^9^ Department of Modern Immunotherapy, Institute of Hematology and Blood Transfusion, Prague, Czechia

**Keywords:** hepcidin, ferritin, sepsis, COVID-19, inflammation, septic shock

## Abstract

**Introduction:**

Major clinically relevant inflammatory events such as septic shock and severe COVID-19 trigger dynamic changes in the host immune system, presenting promising candidates for new biomarkers to improve precision diagnostics and patient stratification. Hepcidin, a master regulator of iron metabolism, has been intensively studied in many pathologies associated with immune system activation, however these data have never been compared to other clinical settings. Thus, we aimed to reveal the dynamics of iron regulation in various clinical settings and to determine the suitability of hepcidin and/or ferritin levels as biomarkers of inflammatory disease severity.

**Cohorts:**

To investigate the overall predictive ability of hepcidin and ferritin, we enrolled the patients suffering with three different diagnoses – in detail 40 patients with COVID-19, 29 patients in septic shock and eight orthopedic patients who were compared to nine healthy donors and all cohorts to each other.

**Results:**

We showed that increased hepcidin levels reflect overall immune cell activation driven by intrinsic stimuli, without requiring direct involvement of infection vectors. Contrary to hepcidin, ferritin levels were more strongly boosted by pathogen-induced inflammation – in septic shock more than four-fold and in COVID-19 six-fold in comparison to sterile inflammation. We also defined the predictive capacity of hepcidin-to-ferritin ratio with AUC=0.79 and *P* = 0.03.

**Discussion:**

Our findings confirm that hepcidin is a potent marker of septic shock and other acute inflammation-associated pathologies and demonstrate the utility of the hepcidin-to-ferritin ratio as a predictor of mortality in septic shock, but not in COVID-19.

## Background

Major inflammatory events such as sepsis trigger significant changes in host metabolism aimed at supporting innate immunity while reducing the ability of pathogens to replicate and spread throughout the body. A particularly important example is iron homeostasis. Iron plays a vital role in host metabolism as a cofactor for enzymes involved in cell respiration, proliferation, and DNA synthesis. Furthermore source of iron is a required by symbiotic commensals as well as most pathogens (1). The sequestering of iron out of circulation (and therefore beyond the reach of extracellular pathogens) is a key mechanism of the innate immune system, and associations between circulating iron levels and various infections have been observed (2). This is primarily true for bacterial, fungal, and parasitic pathogens, but even viral infections may be impacted by iron metabolism; indeed, the association between iron levels and SARS-CoV-2 infection has been intensively studied (3), though further research is needed to fully clarify the functions of iron at the interface between the host immune system and this critical viral pathogen. The accumulation of iron in cells can paradoxically influence intracell pathogens and through increased intracellular availability of iron improve the virulence (4, 5).

Considering the importance of iron in metabolism and immunity, it is unsurprising that the levels of this essential micronutrient are carefully controlled by a system of critical regulatory molecules. Hepcidin is a major iron-regulating peptide hormone that is produced in the liver. Through the control of iron exporter ferroportin, hepcidin plays a central role in iron homeostasis, because it regulates the release of iron to bloodstream from its reservoirs including enterocytes, hepatocytes and iron recycling from erythrocytes (6–9).

Hepcidin expression is normally regulated in a negative feedback loop by systemic levels of iron (7, 10). However, under inflammatory conditions, hepcidin expression can also be induced independently of iron levels through TLR4 activation by LPS (11) or through the IL-6/gp130/STAT3 signaling axis (12–14). We and others showed that plasma levels of IL-6 significantly positively correlated with progression of sepsis (15) and COVID-19. Hepcidin levels are dramatically elevated during infections, including those responsible for sepsis, thereby reducing the amount of iron available to pathogens and also affecting inflammation (16).

Another important player in iron homeostasis is ferritin, the iron repository protein, which binds free iron to prevent the formation of toxic free radicals (17). Under physiological conditions, ferritin is cytosolic protein and its serum variant arising from damaged cells is released under inflammatory conditions (18). Like hepcidin, ferritin levels in blood stream are also boosted independently of iron status during infection and inflammation in order to modulate the immune response. Ferritin is an acute phase reactant, eliminating the available iron in circulation to deprive pathogens and inhibit their growth (19).

This altered metabolism during infection therefore induces significant changes in the circulating levels of iron and its associated regulatory molecules, providing an opportunity to assess disease progression by monitoring these processes. Persistently elevated serum hepcidin in an inflammatory setting produces hypoferremia, leading to a specific condition dubbed “anemia of inflammation” (20, 21) that is often observed during the progression of sepsis (22). Similar to sepsis, multiple studies have also identified dysregulated levels of iron in COVID-19 patients, portraying low serum iron as a potential predictor of the severity and mortality of this disease (23, 24). Hepcidin itself is able to regulate the levels of ferritin. Even, the distant sequence similarity between the cysteine-rich cytoplasmic tail of the SARS-CoV-2 spike protein and the hepcidin protein (25) indicates the ability of this protein to directly increase ferritin levels (26, 27).

 When the levels of a circulating molecule can be correlated with disease progression and/or outcomes, they can serve as valuable biomarkers that facilitate disease monitoring, diagnosis, and patient stratification. As discussed above, levels of circulating hepcidin and ferritin are boosted during sepsis and septic shock, and these levels can also correlate with the severity of these conditions (28). Meanwhile, in severe respiratory infections such as COVID-19, profound dysregulation of the immune system drives mortality (29, 30), and intense research efforts are ongoing to establish immune-related biomarkers for the severity and prognosis of COVID-19. These comprise both cellular (31) and humoral markers including the role of iron homeostasis (32). The objective of this study was to investigate the expression dynamics of ferritin and hepcidin as potential stratification markers in patients with septic shock or COVID-19, with emphasis on the possible immunomodulatory roles of these proteins in the context of long-term immunological exhaustion (e.g., long-term COVID-19 and post-sepsis syndrome).

## Methods

### Research design

In this cross-sectional study, we enrolled in total 29 patients with septic shock, 40 patients with severe COVID-19, eight orthopedic patients who underwent total hip or knee arthroplasty and nine healthy individuals were enrolled to reveal the role of hepcidin and ferritin. Inclusion and exclusion criteria are specified in the specific cohort sections.

### Patient cohorts

#### Septic shock cohort

In total, we enrolled 29 Caucasian patients fulfilling the criteria of septic shock (33) who were admitted to the intensive care unit (ICU) at St. Anne’s University Hospital Brno. Patients with chronic immunosuppression and those who had received antibiotic therapy for more than two days were excluded. All patients were treated with a standardized therapy according to current recommendations (34). Written informed consent was obtained from all enrolled patients, and all procedures and protocols were approved by the institutional ethics committee of St. Anne’s hospital. Blood samples from each patient were collected at four timepoints: at the morning after admission to the ICU (TP1), at 3-5 days after ICU admission (TP2), upon recovery from acute phase (TP3), and at least 6 months after septic shock onset (TP4).

#### COVID-19 cohort

The COVID-19 cohort consisted of 40 Caucasian patients admitted to the ICU at St. Anne’s University Hospital Brno due to respiratory failure on the basis of pneumonia associated with COVID-19. All patients were mechanically ventilated. Written informed consent was obtained from all enrolled patients or their close relative, and all procedures and protocols were approved by the institutional ethics committee of St. Anne’s hospital. Blood samples were collected from all enrolled patients the morning after ICU admission (TP1).

#### Orthopedic cohort

We enrolled eight orthopedic patients who underwent total hip or knee arthroplasty. Written informed consent was obtained from all enrolled patients, and all procedures and protocols were approved by the institutional ethics committee of St. Anne’s hospital. Blood samples were collected at two timepoints: on the day of surgery before induction of anesthesia (TP0) and on the following day (TP1).

#### Healthy controls and general exclusion criteria

Nine buffy coats from healthy adult blood donors were purchased (Department of Transfusion and Tissue Medicine of the Brno University Hospital, Brno, Czech Republic).

The exclusion criteria for all cohorts enrolled in the study included ongoing chronic immunosuppression therapy, oncological disorders, and age < 18 years.

### Blood sample processing

Heparinized blood samples were processed within two hours of collection. Plasma was collected from the centrifuged samples and immediately frozen and stored at -80°C until use.

### Hepcidin measurement

Plasma hepcidin levels were determined using the Intrinsic Hepcidin IDx ELISA kit (Intrinsic LifeSciences) following the manufacturer’s instructions. In brief, all plasma samples were diluted 1:1 in deionized water. Subsequently, all samples and standards were added to a pre-coated microwell plate and incubated with a biotinylated hepcidin tracer for 60 minutes at room temperature (RT). After washing away unbound tracer, all samples were incubated with streptavidin-HRP conjugate for 30 minutes, followed by another wash to remove unbound streptavidin-HRP. Tetramethylbenzidine substrate was then added to each sample, and the reaction was stopped after 15 minutes by the addition of a stop solution. Samples were measured using a microplate reader Multiscan GO (Thermo Scientific) at 450nm.

### Ferritin measurement

The levels of plasma ferritin were determined using the Human Ferritin DuoSet ELISA (R&D Systems) according to the manufacturer’s instructions. In brief, each sample was incubated in microplates pre-coated with capture antibody for two hours at RT, washed with 0.05% Tween 20 in PBS, and incubated with diluted detection antibody for two hours at RT. Samples were next incubated with Streptavidin-HRP B, followed by another incubation with a 1:1 mixture of H_2_O_2_ and tetramethylbenzidine. Both incubations were for 20 minutes at RT. 2 N H_2_SO_4_ was then added and mixed thoroughly to stop the reaction. Washing was performed between each step. The absorbance of samples was measured at 450nm using a microplate reader with a correction at 540nm.

### Statistical analysis

R software (v 4.0.5) was used for statistical analysis. Data were tested for normal distribution using Shapiro-Wilk test and statistical tests were applied as appropriate. The statistical tests used are specified in the figure legends. Columns represent mean and error bars the SD. The level of statistical significance is indicated as follows: *P < 0.05, **P < 0.01, and ***P < 0.001.

## Results

We hypothesized that plasma hepcidin levels would correlate with the severity of sepsis and COVID-19. To test this hypothesis, we enrolled 29 patients suffering from septic shock and 40 patients with COVID-19. We also enrolled eight orthopedic patients undergoing total hip/knee replacement as a control cohort with non-infectious etiology (sterile surgical trauma) and nine healthy individuals to measure background hepcidin and ferritin levels in the absence of major inflammatory events. Detailed demographic and clinical characteristics of all enrolled cohorts are summarized in **Table 1**. Participant age ranges were significantly different across the four cohorts. We found significant difference between COVID-19 and septic shock cohort, also between septic shock and orthopedic cohorts both in comparison to healthy cohort. We observed significant differences between the COVID-19 and septic shock cohorts in body mass index (BMI), creatinine and C-reactive protein (CRP) levels, sequential organ failure assessment (SOFA) score, and the PaO_2_/FiO_2_ ratio (which represents the severity of respiratory failure, with lower values indicating more severe status).

**Table 1:** Demographic and clinical characteristics of each cohort at TP1.

	Cohort			
**Characteristic**	COVID-19	Septic shock	Orthopedic	Healthy
Recruited patients, n	40	29	8	9
Gender				
Female, n (%)	11 (27.5)	11 (37.9)	4 (50)	4 (44.4)
Male, n (%)	29 (72.5)	18 (62.1)	4 (50)	5 (55.6)
Age	61 (29–82) **	71 (51-89) **^###^**	71 (63-82) **^$$^**	43 (26-66)
BMI	30.3 (20-59)*	26.8 (21-34)	31.6 (22-44)	26.1 (22-32)
SOFA score	8.8 (5-15)**	10.8 (3-17)	–	–
CRP (mg/l)	162.8 (8.1-428.6)***	257.2 (65.4-497.4)	–	–
Lactate (mmol/l)	1.5 (0.6-3.3)	2.1 (0.4-6.7)	–	–
Hemoglobin (g/l)	129.3 (97-174)	121.9 (77-171)	116.8 (88-143)	–
Leukocytes (10^3^/μl)	12.3 (3.5-29.1)	19.9 (3.6-96.8)	10.7 (7.9-12.4)	–
Creatinine (mmol/l)	107.9 (43-441)***	232.1 (50-696)	–	–
Bilirubin (μmol/l)	11.8 (3-28.4)	18.6 (1-92.8)	–	–
PaO_2_/FiO_2_ ratio	92.7 (34-159)***	220.3 (101-382)	–	–
Mech. ventilation, n (%)	40 (100)	26 (89.7)	–	–

Values represent the mean, with the range shown in parentheses, unless otherwise indicated. Asterisks indicate significant differences between the COVID-19 and septic shock cohorts, hashes indicate significant difference between septic shock and healthy cohorts, and dollar shows significant difference between orthopedic and healthy cohorts. All data were tested using the Kruskal-Wallis test with Dunn’s post-hoc test (for multiple comparisons) or the Mann-Whitney test (for two-group comparisons). *P < 0.05, **P < 0.01, ***P < 0.001. BMI, body mass index; CRP, C-reactive protein; SOFA, sequential organ failure assessment. ^####^P < 0.001, ^$$^P < 0.01.

**Figure 1:**
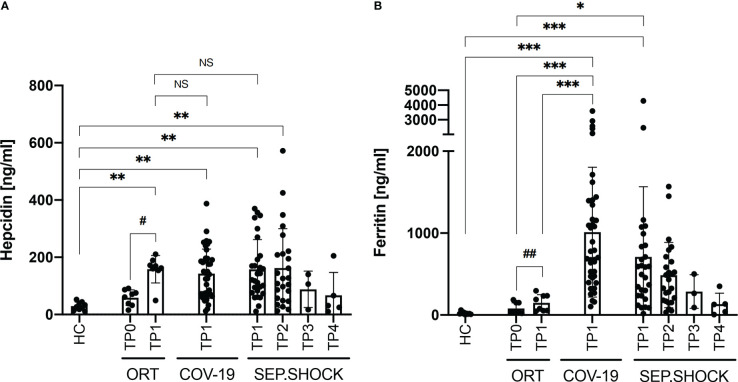
Plasma levels of hepcidin and ferritin under various clinical conditions. **(A)** Plasma hepcidin levels were significantly elevated in all cohorts with inflammatory clinical conditions compared to healthy controls (HC). **(B)** Ferritin levels were significantly boosted in the COVID-19 (COV-19) and septic shock (SEP.SHOCK) cohorts in comparison to the pre-surgery orthopedic cohort (ORT; TP0) and to healthy controls. COVID-19 ferritin levels were also significantly increased in comparison to the post-surgery orthopedic cohort (TP1). Statistical comparisons were performed using the Kruskal-Wallis test with Dunn’s test: *P < 0.05, **P < 0.01, ***P < 0.001, NS = not significant. Paired data (ORT, SEP.SHOCK) were tested using the paired Wilcoxon test: ^#^ *P* < 0.05, ^##^ *P* < 0.01 ^#^*P < 0.05.

We next analyzed the plasma concentrations of hepcidin and ferritin in the cohorts (**Figure 1**), finding significantly elevated levels of hepcidin in the orthopedic, COVID-19, and septic shock cohorts in comparison to the healthy controls (**Figure 1A**). Plasma hepcidin levels were not significantly different among the orthopedic, COVID-19, and septic shock cohorts, suggesting an association between elevated hepcidin and general systemic inflammation. Importantly, we observed a strong significant increase in hepcidin levels in the orthopedic cohort after surgery (TP0 to TP1). In the septic shock cohort, we observed similar levels of hepcidin at TP1 and TP2 (during acute septic shock), followed by a gradual non-significant reduction during recovery from the acute phase (TP3) to the late post-septic shock phase (TP4).

 We also analyzed plasma ferritin levels, finding significantly increased concentrations in the COVID-19 and septic shock cohorts compared to healthy donors (**Figure 1B**). Ferritin levels were also significantly higher in COVID-19 compared the orthopedic cohort both before and after (TP0 and TP1). Similarly, the septic shock cohort showed significantly increased levels of ferritin in comparison to the orthopedic cohort before surgery (TP0) and the healthy controls. That increased ferritin levels were more than six-fold higher in the COVID-19 and more than four-fold higher in the septic shock cohort compared to the non-infectious post-surgery state (TP1) in the orthopedic cohort.

These findings suggested a possible correlation between hepcidin and ferritin levels under inflammatory conditions. We found a significant correlation between the plasma levels of hepcidin and ferritin in the COVID-19 cohort (**Table 2**). In the septic shock and orthopedic cohorts, however, this correlation was not observed.

**Table 2:** Correlation between hepcidin and ferritin levels at TP1 in each cohort.

Cohort	Hepcidin [ng/ml]	Ferritin [ng/ml]	*r* value	*P* value
**COVID-19**	142.96 (12.33-387.17)	1011.91 (102.62-3598.30)	0.518	**< 0.001**
**Septic shock**	158.04 (10.71-369.69)	708.73 (17.55-4282.23)	0.200	0.32
**Orthopedic**	158.43 (49.43-210.56)	148 (46.71-296.33)	0.405	0.33
**Healthy**	29.33 (11.29-52.81)	20.46 (5.77-59.67)	0.467	0.21

Hepcidin and ferritin plasma levels are shown as mean (range) for each cohort. Bolded text = statistically significant correlation. r = Spearman’s correlation coefficient.

**Figure 2:**
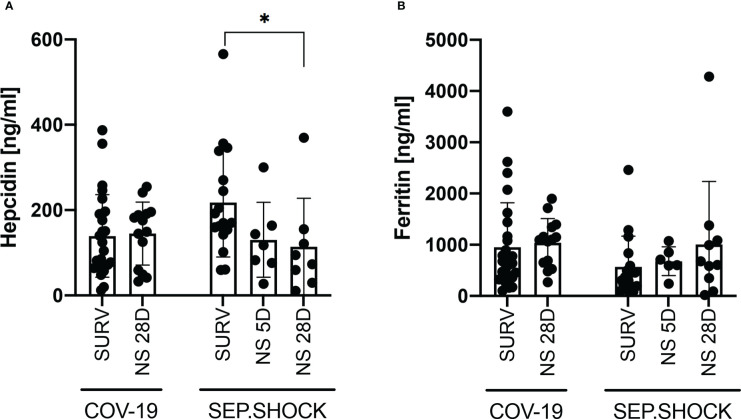
Hepcidin and ferritin levels in infectious disease cohorts (TP1) by patient survival status. **(A, B)** No significant differences in **(A)** hepcidin or **(B)** ferritin levels were observed between survivors and the 28D mortality group in the COVID-19 cohort. In the septic shock cohort, hepcidin levels at TP1 were significantly higher (*P* = 0.023) in survivors than in the 28D mortality group, while the difference between survivors and the 5D mortality group was not significant (*P* = 0.075). **(B)** The mean ferritin level was higher in the 28D mortality group than in the survivor group, but this difference was not significant (*P* = 0.264). Statistical comparisons were performed using the Mann-Whitney test. NS, non-survivor; SURV, survivor. ﻿*P < 0.05.

We subsequently focused on the ability of hepcidin and ferritin levels to predict the prognosis and severity of COVID-19 and the progression of septic shock (**Figure 2**). We evaluated the TP1 plasma hepcidin and ferritin levels in septic shock patients who had died by five days (5D mortality group) or 28 days (28D mortality group) after admission to ICU. On the other hand, only the 28D mortality group was analyzed in the COVID-19 cohort, since no COVID-19 patients had died by the 5D timepoint. There were no significant differences in plasma hepcidin concentrations between survivors and COVID-19 patients who died within the first 28 days after disease onset (**Figure 2A**). In the septic shock cohort, however, the highest mean hepcidin concentrations were in patients who survived the septic shock, and this difference was significant between survivors and the 28D mortality group (**Figure 2A**).

We also did not observe a significant difference in ferritin levels between COVID-19 survivors and the 28D mortality group (**Figure 2B**). In the septic shock cohort, the lowest concentrations of ferritin were found in survivors, but the mean concentration was not significantly different compared to the 5D and 28D mortality groups (**Figure 2B**).

**Figure 3:**
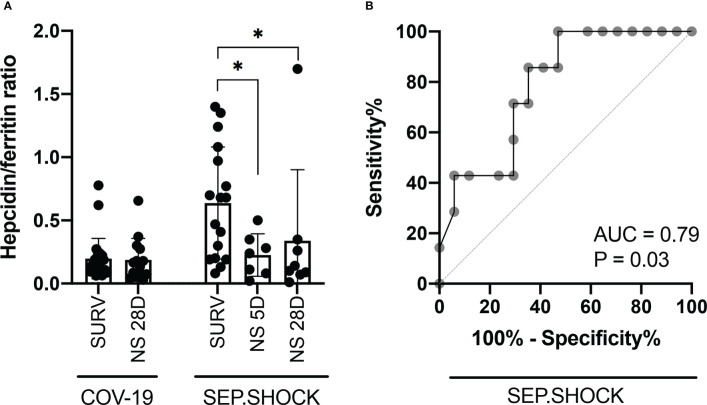
The hepcidin-to-ferritin ratio is a potential marker for stratification of septic shock patients. **(A)** The TP1 hepcidin-to-ferritin ratio was significantly lower in the 5D and 28D mortality groups in comparison to survivors in the septic shock cohort. **(B)** ROC curve analysis was performed to evaluate the diagnostic potential of the hepcidin-to-ferritin ratio for predicting overall mortality in septic shock patients. The resulting AUC was 0.79, indicating a predictive potential. Statistical comparisons were performed using the Mann-Whitney test. AUC, area under the curve; NS, non-survivor; ROC, receiver operating characteristic; SURV, survivor. *P < 0.05.

Based on the expression dynamics of hepcidin and ferritin in the septic shock cohort, we evaluated the hepcidin-to-ferritin ratio to better reflect the overall prognosis of the participants in this cohort, thus pinpoint those vulnerable patients with the need of meticulous medical supervision. We observed a significantly lower hepcidin-to-ferritin ratio in both mortality groups compared to the survivors in this cohort (**Figure 3A**), although this ratio did not show a significant trend between survivors and non-survivors in the COVID-19 cohort. Subsequently, we also confirmed the predictive potential of the hepcidin-to-ferritin ratio for overall mortality in the septic shock cohort by receiver operating characteristics (ROC) analysis, finding that this marker displayed significant predictive potential with an area under the ROC curve (AUC) of 0.79 (**Figure 3B**).

We next investigated the possibility of correlations between hepcidin or ferritin levels and the length of survival in the non-survivors within the COVID-19 and septic shock cohorts (**Table 3**). We found a significant positive correlation between ferritin levels and days of survival in the COVID-19 cohort. No other parameter displayed a correlation with days of patient survival. Finally, we explored whether these marker levels correlated with SOFA scores in the COVID-19 and septic shock patients (**Table 4**), finding a weak negative correlation between SOFA score and ferritin levels in the former cohort.

**Table 3:** Correlation between TP1 hepcidin or ferritin levels and days survived by deceased infectious disease patients.

			Hepcidin		Ferritin	
**Cohort**	**Deceased, n (%)**	**Mean days of survival (range)**	***r* value**	***P* value**	***r* value**	***P* value**
**COVID-19**	17 (42.5%)	16 (4-29)	0.295	0.25	0.54	**0.024**
**Septic shock**	17 (58.62%)	31 (0-211)	-0.08	0.77	0.04	0.88

Bolded text = statistically significant correlation. r, Spearman correlation coefficient.

**Table 4:** Correlation between TP1 hepcidin or ferritin levels and SOFA scores of all enrolled patients.

		Hepcidin		Ferritin	
**Cohort**	**SOFA score (mean)**	***r* value**	***P* value**	***r* value**	***P* value**
**COVID-19**	9 (5-15)	-0.02	0.90	-0.31	**0.05**
**Septic shock**	11.2 (3-17)	0.13	0.50	0.28	0.16

r, Spearman correlation coefficient; SOFA, sequential organ failure assessment. Bolded text = statistically significant correlation.

## Discussion

In this study, we focused on hepcidin and ferritin as markers of septic shock and COVID-19 and tested their predictive potential for disease severity. Hepcidin and ferritin dynamics in these infectious disease cohorts were compared with the orthopedic cohort, where major surgery (hip or knee replacement) led to immune system activation without pathogenic stimuli. This condition, where sterile inflammation or systemic inflammatory response syndrome (SIRS)-like occurs early after surgery, helped us to determine whether the observed changes in hepcidin levels were pathogen-dependent or -independent.

The role of iron homeostasis in COVID-19 has recently been investigated (32), with a significant strong association found between decreased iron levels and COVID-19 progression. We have also previously shown that survival of fulminant septic shock can be associated with the specific proteomic profile of neutrophils (35). One of the involved proteins was lactotransferrin, an iron-binding protein. The neutrophil-to-CD8^+^ T cell ratio in severe COVID-19 patients was also found to predict in-ICU mortality, thereby stratifying patients and identifying those more susceptible to the disease (31). Neutrophils are one of the key immune cell subsets in sepsis-associated dysregulation of the immune system as well (36), and iron levels have been shown to have a profound impact on neutrophil functionality (37). Altogether, these findings suggest that dysregulated iron and hepcidin levels may be related to disease-associated neutrophil dysfunction.

The role of hepcidin and the predictive ability of its circulating concentrations during COVID-19 and septic shock remain controversial. An analysis of biomarkers in COVID-19 patients showed that serum iron levels were strongly associated with IL-6 and other inflammatory components, but no correlation was observed with COVID-19 recovery or even hepcidin levels (32). Conversely, other groups have associated higher levels of hepcidin or iron with the severity of COVID-19 (38–41) or sepsis (22), though studies in mice have reported that high hepcidin levels play a beneficial role in sepsis models (42–44). Similarly, several neonatal studies have shown a protective role for elevated hepcidin against various bacterial infections in children (45, 46). In the context of these data, it is clear that we and others confirm increased levels of hepcidin during severe inflammatory/infectious events such as septic shock, COVID-19, and the SIRS-like post-surgery inflammatory status.

While significant changes in hepcidin levels have been reported during infectious events such as sepsis and COVID-19, our results show for the first time that circulating hepcidin also reflects the activation of the immune system in the absence of invading pathogens. This finding contrasts with previous data showing a correlation between hepcidin expression and the infection state in a patient with epididymitis and sepsis, suggesting a direct effect of bacteria on hepcidin levels (47). Similarly, other studies reported that hepcidin levels decreased during the course of successful patient antibiotic therapy (48, 49). Based on our data, we speculate that this antibiotic therapy instead had an *indirect* effect on the observed gradual decrease in hepcidin levels, since hepcidin expression was also induced by non-pathogenic intrinsic stimuli and persisted at increased levels long after the period when patients were treated with antibiotics.

Interestingly, we observed similar levels of hepcidin in the COVID-19, septic shock, and orthopedic cohorts during TP1, indicating a stronger influence by general inflammatory mediators and overall immune dysregulation than by specific infectious vectors. Contrary to hepcidin, we found that ferritin levels were six-fold and four-fold higher in the COVID-19 and septic shock cohorts, respectively, in comparison to the orthopedic cohort. Although ferritin has previously been found to reflect the development of severe complications in COVID-19 (50), we did not observe any significant difference in the levels of hepcidin or ferritin between COVID-19 survivors and 28D non-survivors.

We subsequently analyzed the hepcidin-to-ferritin ratio, which showed a significant ability to predict 5D and 28D mortality in septic shock patients. It was previously reported that, in some clinical settings, the expression of hepcidin and ferritin can be fully driven by inflammation and not only by the regular need to mediate iron metabolism (7). During inflammation, elevation of hepcidin and the subsequent reduction in iron availability lead to impaired hemoglobin synthesis, followed by the development of anemia of inflammation (16). Moreover, under inflammatory conditions, rapid synthesis of ferritin leads to the generation of ferritin with a significantly lower amount of bound iron than in healthy conditions and even more contributing to the anemia of inflammation (51, 52). In COVID-19, meanwhile, the overall condition is also affected by the hepcidin-mimicking spike protein of SARS-CoV-2, which can increase ferritin levels independently of inflammatory stimuli (27). This could explain progressive anemia and hyperferritinemia in COVID-19 (53, 54).

Our study showed that the levels of hepcidin and ferritin at TP1 behaved differently in the septic shock and COVID-19 cohorts: in the former, the expression of both proteins was independent, while in the latter, their expression levels were positively correlated. These data indicate that, although COVID-19 and septic shock share similar illness manifestations such as cytokine storm, there are still differences in illness etiology and progression that likely influence hepcidin and ferritin levels. Therefore, although associations between hepcidin or ferritin levels and disease severity have been observed multiple times (22, 38–41), they cannot be used universally as predictors for mortality. However, hepcidin remains a reliable marker of early sepsis onset due to its expression kinetics, with elevated levels observable early after induction by various pathogenic and non-pathogenic stimuli. Other iron indicators can be used to validate the importance of iron, especially transferrin as it`s levels can be also affected during infection or inflammation. In COVID-19 infection Claise et al. have shown that transferrin levels were decreased during severe infection in comparison to boosted levels of ferritin (55), this corroborates our ferritin levels observations.

The etiology and immune mechanisms underlying inflammatory disease connects with use of hepcidin and ferritin as markers for predicting mortality. It was previously reported that the expression of hepcidin was induced mainly by the IL-6 signaling. Notably, adipocytes are an important source of IL-6 (56), and blocking of the IL-6 axis leads to the accumulation of adipose tissue mass and subsequently increased IL-6 production (57). Our study revealed a significant difference in BMI between the COVID-19 and septic shock cohorts at TP1, correlating with changes in IL-6 which we have reported earlier in sepsis cohorts (15). Our data also indirectly confirmed one of the hallmarks of organism aging – namely, we observed higher hepcidin levels in the elderly orthopedic cohort in comparison to the younger healthy controls (HC). We suggest that a role of chronic low-grade inflammation in hepcidin induction should be considered in future research, since IL-6 is a well-known player in low-grade inflammation. Indeed, dysbiosis needs to be taken into consideration also in the context of chronic inflammation (58–60) and its contribution to hepcidin production. Microbiota can modulate many mutual processes within host (61, 62), and its composition is heavily affected during COVID-19 or sepsis, where can represents source of further stimulation molecules leading to endotoxemia. The complex mutual mechanism may provide multiple targets to take therapeutical advantage for better infection control.

**Figure 4:**
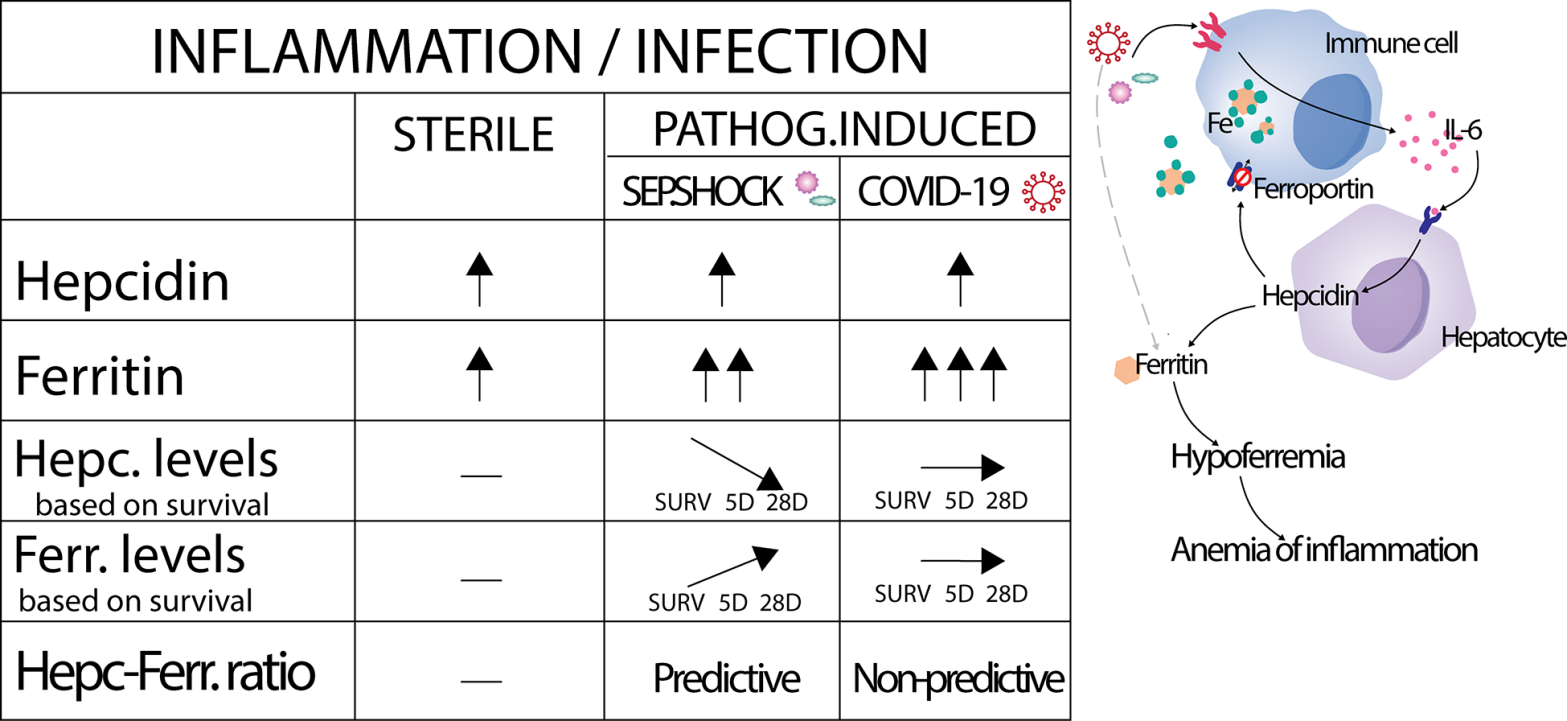
Graphical summarization of obtained results. Activation by sterile inflammation, but also by pathogens lead to increased production of hepcidin at the same manner. Subsequently, the same activation leads to pathogen dependent production of ferritin. In septic shock had levels of hepcidin and ferritin inverse dynamics based on survivorship and led us to prepare hepcidin-ferritin ratio with capacity to predict the prognosis of septic shock patients.

In summary (**Figure 4**), we report here that hepcidin levels were significantly increased during the onset of septic shock, in severe COVID-19, and shortly after major surgery, demonstrating its value as a very early marker of immune system activation in pathogenic and non-pathogenic inflammatory contexts. Corresponding increases in ferritin appeared to be more specific for different causes of inflammation onset: ferritin levels clearly increased in the septic shock and COVID-19 cohorts compared to healthy controls, but only a small significant increase was observed after surgery in the orthopedic cohort. Importantly, we have established a significant diagnostic value of the hepcidin-to-ferritin ratio for predicting mortality in septic shock patients, while this ratio failed to predict the prognosis of COVID-19 in our tested cohort. These findings confirm hepcidin as a potent marker of septic shock and of other acute inflammatory pathologies under certain conditions, and we believe that hepcidin can serve as valuable marker in various clinical settings. Nevertheless, this study has several limitations, namely the number of enrolled patients in all cohorts recruited from single center, or numerous comorbidities of enrolled patients, which can influence the levels of hepcidin and ferritin. Based on these limitations, validation of our data will be required. Future studies will be necessary to define how the observed discrepancies in hepcidin dynamics are driven within different inflammatory and pathological contexts.

## Data availability statement

The raw data supporting the conclusions of this article will be made available by the authors, without undue reservation.

## Ethics statement

The studies involving human participants were reviewed and approved by The institutional ethics committee of St. Anne’s hospital. The patients/participants provided their written informed consent to participate in this study.

## Author contributions

MH-K: Formal analysis, Investigation, Data analysis, Visualization, Writing – Original Draft, Writing – Review and Editing. MSk: Methodology, Formal analysis, Investigation, Data curation, Visualization. IO: Conceptualization, Methodology, Investigation. MSl, RP, LO, PS, MDZ, OM, IA, PL, VO, MDZ, AM, MŠ, MV: Investigation. VŠ, MH, RP, MD, LO: Patient inclusion/exclusion strategy, Patient recruitment, Clinical data collection. GS: Conceptualization, Supervision, Funding acquisition. RP: Funding acquisition. MH: Conceptualization, Writing – Review and Editing. JF: Conceptualization, Supervision, Project administration, Funding acquisition, Writing – Original Draft, Writing – Review and Editing. All authors contributed to the article and approved the submitted version.

## References

[1] Nairz M Weiss G. Iron in infection and immunity. Mol Aspects Med (2020) 75:1–18. doi: 10.1016/j.mam.2020.100864 32461004

[2] Drakesmith H Prentice AM. Hepcidin and the iron-infection axis. Science (2012) 338(6108):768–72. doi: 10.1126/science.1224577 23139325

[3] Girelli D Marchi G Busti F Vianello A. Iron metabolism in infections: Focus on COVID-19. Semin Hematol (2021) 58(3):182–7. doi: 10.1053/j.seminhematol.2021.07.001 PMC8305218 34389110

[4] Leon-Sicairos N Reyes-Cortes R Guadrón-Llanos AM Madueña-Molina J Leon-Sicairos C Canizalez-Román A. Strategies of intracellular pathogens for obtaining iron from the environment. BioMed Res Int (2015) 1–17. doi: 10.1155/2015/476534 PMC4450229 26120582

[5] Yan Q Zhang W Lin M Teymournejad O Budachetri K Lakritz J. Iron robbery by intracellular pathogen via bacterial effector-induced ferritinophagy. Proc Natl Acad Sci U.S.A. (2021) 118(23):e2026598118. doi: 10.1073/pnas.2026598118 34074773 PMC8201858

[6] Melnick L A Sheng MC. Ironing out ferroportin. Physiol Behav (2016) 176(1):100–6. doi: 10.1016/j.cmet.2015.09.006

[7] Ganz T. Systemic iron homeostasis. Physiol Rev (2013) 93(4):1721–41. doi: 10.1152/physrev.00008.2013 24137020

[8] Tan TCH Crawford DHG Franklin ME Jaskowski LA Macdonald GA Jonsson JR. The serum hepcidin:ferritin ratio is a potential biomarker for cirrhosis. Liver Int (2012) 32(9):1391–9. doi: 10.1111/j.1478-3231.2012.02828.x 22676252

[9] Seyoum Y Baye K Humblot C. Iron homeostasis in host and gut bacteria - a complex interrelationship. Gut Microbes (2021) 13(1):1–19. doi: 10.1080/19490976.2021.1874855 PMC7872071 33541211

[10] Nicolas G Chauvet C Viatte L Danan JL Bigard X Devaux I. The gene encoding the iron regulatory peptide hepcidin is regulated by anemia, hypoxia, and inflammation. J Clin Invest. (2002) 110(7):1037–44. doi: 10.1172/JCI0215686 PMC151151 12370282

[11] Pigeon C Ilyin G Courselaud B Leroyer P Turlin B Brissot P. A new mouse liver-specific gene, encoding a protein homologous to human antimicrobial peptide hepcidin, is overexpressed during iron overload. J Biol Chem (2001) 276(11):7811–9. doi: 10.1074/jbc.M008923200 11113132

[12] Nemeth E Tuttle MS Powelson J Vaughn MD Donovan A Ward DMV. Hepcidin regulates cellular iron efflux by binding to ferroportin and inducing its internalization. Science (2004) 306(5704):2090–3. doi: 10.1126/science.1104742 15514116

[13] Wessling-Resnick M. Iron homeostasis and the inflammatory response. Annu Rev Nutr (2010) 30:105–22. doi: 10.1146/annurev.nutr.012809.104804 PMC3108097 20420524

[14] Camaschella C Nai A Silvestri L. Iron metabolism and iron disorders revisited in the hepcidin era. Haematologica (2020) 105(2):260–72. doi: 10.3324/haematol.2019.232124 PMC7012465 31949017

[15] Hortová-Kohoutková M Lázničková P Bendíčková K De Zuani M Andrejčinová I Tomášková V. Differences in monocyte subsets are associated with short-term survival in patients with septic shock. J Cell Mol Med (2020) 24(21):12504–12. doi: 10.1111/jcmm.15791 PMC7686971 32949213

[16] Wang CY Babitt JL. Hepcidin regulation in the anemia of inflammation. Curr Opin Hematol (2016) 23(3):189–97. doi: 10.1097/MOH.0000000000000236 PMC4993159 26886082

[17] Kernan KF Carcillo JA. Hyperferritinemia and inflammation. Int Immunol (2017) 29(9):401–9. doi: 10.1093/intimm/dxx031 PMC5890889 28541437

[18] Kell DB Pretorius E. Serum ferritin is an important inflammatory disease marker, as it is mainly a leakage product from damaged cells. Metallomics. (2014) 6(4):748–73. doi: 10.1039/C3MT00347G 24549403

[19] Thachil J. The beneficial effect of acute phase increase in serum ferritin. Eur J Intern Med (2016) 35:e16–7. doi: 10.1016/j.ejim.2016.07.020 27491588

[20] Liu Q Davidoff O Niss K Haase VH. Hypoxia-inducible factor regulates hepcidin *via* erythropoietin-induced erythropoiesis. J Clin Invest. (2012) 122(12):4635–44. doi: 10.1172/JCI63924 PMC3533545 23114598

[21] Weiss G Ganz T Goodnough LT. Anemia of inflammation. Blood. (2019) 133(1):40–50. doi: 10.1182/blood-2018-06-856500 30401705 PMC6536698

[22] Jiang Y Jiang FQ Kong F An MM Jin BB Cao D. Inflammatory anemia-associated parameters are related to 28-day mortality in patients with sepsis admitted to the ICU: a preliminary observational study. Ann Intensive Care (2019) 9(1):1–11. doi: 10.1186/s13613-019-0542-7 PMC6557959 31183575

[23] Zhao K Huang J Dai D Feng Y Liu L Nie S. Serum iron level as a potential predictor of coronavirus disease 2019 severity and mortality: A retrospective study. Open Forum Infect Dis (2020) 7(7):1–8. doi: 10.1093/ofid/ofaa250 PMC7337740 32661499

[24] Hippchen T Altamura S Muckenthaler MU Merle U. Hypoferremia is associated with increased hospitalization and oxygen demand in COVID-19 patients. HemaSphere. (2020) 4(6):1–9. doi: 10.1097/HS9.0000000000000492 PMC7665253 33205000

[25] Ehsani S. COVID-19 and iron dysregulation: distant sequence similarity between hepcidin and the novel coronavirus spike glycoprotein. Biol Direct. (2020) 15(1):1–13. doi: 10.1186/s13062-020-00275-2 33066821 PMC7563913

[26] Garrick MD Ghio AJ. Iron chelation may harm patients with COVID-19. Eur J Clin Pharmacol (2021) 77(2):265–6. doi: 10.1007/s00228-020-02987-w PMC7459091 32870379

[27] Abobaker A. Reply: Iron chelation may harm patients with COVID-19. Eur J Clin Pharmacol (2021) 77(2):267. doi: 10.1007/s00228-020-02988-9 32870381 PMC7459945

[28] Tacke F Nuraldeen R Koch A Strathmann K Hutschenreuter G Trautwein C. Iron parameters determine the prognosis of critically ill patients. Crit Care Med (2016) 44(6):1049–58. doi: 10.1097/CCM.0000000000001607 26934143

[29] Giamarellos-Bourboulis EJ Netea MG Rovina N Akinosoglou K Antoniadou A Antonakos N. Complex immune dysregulation in COVID-19 patients with severe respiratory failure. Cell Host Microbe (2020) 27(6):992–1000.e3. doi: 10.1016/j.chom.2020.04.009 32320677 PMC7172841

[30] Schultze JL Aschenbrenner AC. COVID-19 and the human innate immune system. Cell (2021) 184(7):1671–92. doi: 10.1016/j.cell.2021.02.029 PMC7885626 33743212

[31] De Zuani M Lazničková P Tomašková V Dvončová M Forte G Stokin GB. High CD4-to-CD8 ratio identifies an at-risk population susceptible to lethal COVID-19. Scand J Immunol (2022) 95(3):1–11. doi: 10.1111/sji.13125 PMC9286348 34861051

[32] Frost JN Hamilton F Arnold D Elvers KT Shah A Armitage AE. Evaluation of perturbed iron-homeostasis in a prospective cohort of patients with COVID-19. Wellcome Open Res (2022) 7:173. doi: 10.12688/wellcomeopenres.17904.1 35935705 PMC9307999

[33] Singer M Deutschman CS Seymour CW Shankar-Hari M Annane D Bauer M. The third international consensus definitions for sepsis and septic shock (Sepsis-3). Am Med Assoc (2016), 315(8):801–10. doi: 10.1001/jama.2016.0287 PMC4968574 26903338

[34] Rhodes A Evans LE Alhazzani W Levy MM Antonelli M Ferrer R. Surviving sepsis campaign: International guidelines for management of sepsis and septic shock: 2016. Crit Care Med (2017) 45:486–552. doi: 10.1097/CCM.0000000000002255 28098591

[35] Hortová-Kohoutková M De Zuani M Lázničková P Bendíčková K Mrkva O Andrejčinová I. Polymorphonuclear cells show features of dysfunctional activation during fatal sepsis. Front Immunol (2021) 12. doi: 10.3389/fimmu.2021.741484 PMC8710474 34966382

[36] Vymazal O Bendíčková K De Zuani M Vlková M Hortová-Kohoutková M Frič J. Immunosuppression affects neutrophil functions: Does calcineurin-NFAT signaling matter? Front Immunol (2021) 12:4629. doi: 10.3389/fimmu.2021.770515 PMC8593005 34795676

[37] Renassia C Louis S Cuvellier S Boussetta N Deschemin JC Borderie D. Neutrophils from hereditary hemochromatosis patients are protected from iron excess and are primed. Blood Adv (2020) 4(16):3853–63. doi: 10.1182/bloodadvances.2020002198 PMC7448602 32810223

[38] Zhou C Chen Y Ji Y He X Xue D. Increased serum levels of hepcidin and ferritin are associated with severity of COVID-19. Med Sci Monit (2020) 26:1–6. doi: 10.12659/MSM.926178 PMC7526336 32978363

[39] Moreira AC Teles MJ Silva T Bento CM Alves IS Pereira L. Iron related biomarkers predict disease severity in a cohort of Portuguese adult patients during COVID-19 acute infection. Viruses (2021) 13:2482. doi: 10.3390/v13122482 34960751 PMC8703662

[40] Nai A Lorè NI Pagani A De Lorenzo R Di Modica S Saliu F. Hepcidin levels predict covid-19 severity and mortality in a cohort of hospitalized Italian patients. Am J Hematol (2021) 96(1):E32–5. doi: 10.1002/ajh.26027 33075189

[41] Chakurkar V Rajapurkar M Lele S Mukhopadhyay B Lobo V Injarapu R. Increased serum catalytic iron may mediate tissue injury and death in patients with COVID-19. Sci Rep [Internet]. (2021) 11(1):1–8. doi: 10.1038/s41598-021-99142-x 34608227 PMC8490366

[42] Scindia Y Wlazlo E Leeds J Loi V Ledesma J Cechova S. Protective role of hepcidin in polymicrobial sepsis and acute kidney injury. Front Pharmacol (2019) 615. doi: 10.3389/fphar.2019.00615 PMC6563000 31244655

[43] Zeng C Chen Q Zhang K Chen Q Song S Fang X. Hepatic hepcidin protects against polymicrobial sepsis in mice by regulating host iron status. Anesthesiology (2015) 122(2):374–86. doi: 10.1097/ALN.0000000000000466 25264597

[44] Stefanova D Raychev A Deville J Humphries R Campeau S Ruchala P. Hepcidin protects against lethal escherichia coli sepsis in mice inoculated with isolates from septic patients. Infect Immun (2018) 86(7):1–12. doi: 10.1128/IAI.00253-18 PMC6013672 29735522

[45] Prentice S Jallow AT Sinjanka E Jallow MW Sise EA Kessler NJ. Hepcidin mediates hypoferremia and reduces the growth potential of bacteria in the immediate post-natal period in human neonates. Sci Rep (2019) 9(1):1–7. doi: 10.1038/s41598-019-52908-w 31719592 PMC6851364

[46] Abuga KM Muriuki JM Uyoga SM Mwai K Makale J Mogire RM. Hepcidin regulation in Kenyan children with severe malaria and non-typhoidal salmonella bacteremia. Haematologica (2021) 107(7). doi: 10.3324/haematol.2021.279316 PMC9244826 34498446

[47] Nemeth E Valore EV Territo M Schiller G Lichtenstein A Ganz T. Hepcidin, a putative mediator of anemia of inflammation, is a type II acute-phase protein. Blood. (2003) 101(7):2461–3. doi: 10.1182/blood-2002-10-3235 12433676

[48] Elgendy FM Khatab AA Badr HS Fatah G Fishawy AM El. Evaluation of hepcidin as a biomarker for neonatal sepsis. Menoufia Med J (2018) 31(3):977. doi: 10.4103/mmj.mmj_32_17

[49] Olinder J Ehinger D Liljenborg E Herwald H Rydén C. Plasma levels of hepcidin and reticulocyte haemoglobin during septic shock. J Innate Immun (2020) 12(6):448–60. doi: 10.1159/000508561 PMC7747076 32950976

[50] Carubbi F Salvati L Alunno A Maggi F Borghi E Mariani R. Ferritin is associated with the severity of lung involvement but not with worse prognosis in patients with COVID-19: data from two Italian COVID-19 units. Sci Rep (2021) 11(1):1–11. doi: 10.1038/s41598-021-83831-8 33649408 PMC7921386

[51] Ten Kate J Drenth JPH Kahn MF Van Deursen C. Iron saturation of serum ferritin in patients with adult onset still’s disease. J Rheumatol (2001) 28(10):2213–5. doi: 10.1002/art.21164 11669158

[52] Herbert V Jayatilleke E Shaw S Rosman AS Giardina P Grady RW. Serum ferritin iron, a new test, measures human body iron stores unconfounded by inflammation. Stem Cells (1997) 15(4):291–6. doi: 10.1002/stem.150291 9253113

[53] Cavezzi A Troiani E Corrao S. COVID-19: Hemoglobin, iron, and hypoxia beyond inflammation. A Narrative Review. Clin Pract (2020) 10(2):24–30. doi: 10.4081/cp.2020.1271 PMC7267810 32509258

[54] Perricone C Bartoloni E Bursi R Cafaro G Guidelli GM Shoenfeld Y. COVID-19 as part of the hyperferritinemic syndromes: the role of iron depletion therapy. Immunol Res (2020) 68(4):213–24. doi: 10.1007/s12026-020-09145-5 PMC7366458 32681497

[55] Claise C Saleh J Rezek M Vaulont S Peyssonnaux C Edeas M. Low transferrin levels predict heightened inflammation in patients with COVID-19: New insights. Int J Infect Dis (2022) 116:74–9. doi: 10.1016/j.ijid.2021.12.340 PMC8688186 34952211

[56] Han MS White A Perry RJ Camporez JP Hidalgo J Shulman GI. Regulation of adipose tissue inflammation by interleukin 6. Proc Natl Acad Sci U.S.A. (2020) 117(6):2751–60. doi: 10.1073/pnas.1920004117 PMC7022151 31980524

[57] Sindhu S Thomas R Shihab P Sriraman D Behbehani K Ahmad R. Obesity is a positive modulator of IL-6R and IL-6 expression in the subcutaneous adipose tissue: Significance for metabolic inflammation. PloS One (2015) 10(7):e0133494. doi: 10.1371/journal.pone.0133494 26200663 PMC4511728

[58] Zeng MY Inohara N Nuñez G. Mechanisms of inflammation-driven bacterial dysbiosis in the gut. Mucosal Immunol (2017) 10(1):18–26. doi: 10.1038/mi.2016.75 27554295 PMC5788567

[59] van den Munckhof ICL Kurilshikov A ter Horst R Riksen NP Joosten LAB Zhernakova A. Role of gut microbiota in chronic low-grade inflammation as potential driver for atherosclerotic cardiovascular disease: a systematic review of human studies. Obes Rev (2018) 19(12):1719–34. doi: 10.1111/obr.12750 30144260

[60] Xu YS Liu XJ Liu XX Chen D Wang MM Jiang X. The roles of the gut microbiota and chronic low-grade inflammation in older adults with frailty. Front Cell Infect Microbiol (2021) 11:586. doi: 10.3389/fcimb.2021.675414 PMC8282182 34277468

[61] Das NK Schwartz AJ Barthel G Inohara N Liu Q Sankar A. Microbial metabolite signaling is required for systemic iron homeostasis. Cell Metab (2020) 31(1):115–130.e6. doi: 10.1016/j.cmet.2019.10.005 31708445 PMC6949377

[62] Bessman NJ Mathieu JRR Renassia C Zhou L Fung TC Fernandez KC. Dendritic cell-derived hepcidin sequesters iron from the microbiota to promote mucosal healing. Science (2020) 368(6487):186–9. doi: 10.1126/science.aau6481 PMC7724573 32273468

